# A new direction for prenatal chromosome microarray testing: software-targeting for detection of clinically significant chromosome imbalance without equivocal findings

**DOI:** 10.7717/peerj.354

**Published:** 2014-04-22

**Authors:** Joo Wook Ahn, Susan Bint, Melita D. Irving, Phillipa M. Kyle, Ranjit Akolekar, Shehla N. Mohammed, Caroline Mackie Ogilvie

**Affiliations:** 1 Cytogenetics, Guy’s & St Thomas’ NHS Foundation Trust, London, UK; 2 Cytogenetics, GSTS Pathology, London, UK; 3 Clinical Genetics, Guy’s & St Thomas’ NHS Foundation Trust, London, UK; 4 Fetal Medicine Unit, Guy’s & St Thomas’ NHS Foundation Trust, London, UK; 5 Fetal Medicine Unit, Medway Maritime Hospital, Kent, UK; 6 King’s College, London, UK

**Keywords:** Array CGH, CMA, Software targeting, Prenatal, VUS, Prenatal testing

## Abstract

**Purpose.** To design and validate a prenatal chromosomal microarray testing strategy that moves away from size-based detection thresholds, towards a more clinically relevant analysis, providing higher resolution than G-banded chromosomes but avoiding the detection of copy number variants (CNVs) of unclear prognosis that cause parental anxiety.

**Methods.** All prenatal samples fulfilling our criteria for karyotype analysis (*n* = 342) were tested by chromosomal microarray and only CNVs of established deletion/duplication syndrome regions and any other CNV >3 Mb were detected and reported. A retrospective full-resolution analysis of 249 of these samples was carried out to ascertain the performance of this testing strategy.

**Results.** Using our prenatal analysis, 23/342 (6.7%) samples were found to be abnormal. Of the remaining samples, 249 were anonymized and reanalyzed at full-resolution; a further 46 CNVs were detected in 44 of these cases (17.7%). None of these additional CNVs were of clear clinical significance.

**Conclusion.** This prenatal chromosomal microarray strategy detected all CNVs of clear prognostic value and did not miss any CNVs of clear clinical significance. This strategy avoided both the problems associated with interpreting CNVs of uncertain prognosis and the parental anxiety that are a result of such findings.

## Introduction

Implementation of chromosomal microarray analysis (CMA) for prenatal testing continues to be debated and no international consensus has been reached. The problems, challenges and advantages of CMA have been extensively discussed in the literature (Evangelidou et al., 2013; Ganesamoorthy et al., 2013; Vetro et al., 2012; Wapner et al., 2012) as well as at conference debates (Crolla, Wapner & Van Lith, 2013); several centers have already implemented this powerful tool for investigation of prenatal samples (usually following detection of a fetal anomaly by ultrasound) (Evangelidou et al., 2013; Fiorentino et al., 2011; Liao et al., 2013; Srebniak et al., 2011). We feel that the advantage of increased resolution offered by CMA over G-banded karyotype analysis must be balanced against the potential distress and anxiety caused to a couple by reporting CNVs of unknown significance, and/or incidental findings of clinical significance but of no relevance to the fetal abnormalities. This stance holds particularly true within the context of a state-funded healthcare system such as that in the UK, where funds are limited and there is an emphasis on clinical and cost effectiveness of any diagnostic test, within the context of the whole healthcare service. Therefore, this assessment necessarily extends beyond the test itself and includes, for example, the increased requirement for genetic counseling that can be substantial when variants of uncertain clinical significance are detected. A recent survey of prenatal genetic counselors showed that the majority (57%) were uncomfortable helping patients decide about pregnancy termination following uncertain results (Bernhardt et al., 2014). This is not the only study to highlight the issues around unclear results and to suggest that more training and education is required (Mikhaelian et al., 2013).

We have performed close to 20,000 diagnostic postnatal CMA tests at our center (at a median resolution of 120 kb) and reported CNVs of unclear clinical significance for 10% of those cases (Ahn et al., 2013); a recent prenatal CMA study using a similar platform reported 8.4% (Hillman et al., 2013), while another prenatal CMA study with a lower resolution platform (200 kb) reported 6.7% (Ganesamoorthy et al., 2013). These figures illustrate the potential volume of uncertain results following prenatal CMA.

Different models have been proposed to maximize the benefits of prenatal CMA whilst addressing concerns regarding results with uncertain prognoses; the most widespread approach being to test only those pregnancies with fetal abnormalities on ultrasound (Stark et al., 2013), although application to all those having invasive testing has also been suggested (Brady et al., 2013). In concert with limiting access to prenatal CMA, many laboratories are limiting the diagnostic potential of the test by using a targeted platform (Shaffer et al., 2008) or a lower resolution platform (Hillman et al., 2013); both of these strategies were designed to reduce ambiguous findings. Conversely, some laboratories have reported application of whole-genome arrays (Faas et al., 2010) having decided in favor of prioritizing detection of all CNVs of potential clinical significance over the issues associated with variants of unknown significance. Some centers have set up committees for detailed scrutiny of such findings in order to arrive at a consensus on if and how to report them (Liao et al., 2013; Vetro et al., 2012); (http://www.nets.nihr.ac.uk/projects/eme/106003).

Our center has taken the position that prenatal CMA should present couples with results of clear clinical significance. Our prenatal CMA platform therefore has a 3 Mb backbone resolution and ∼120 kb resolution in specific regions of known clinical significance (see Table 1). Furthermore, we have decoupled the diagnostic resolution of the test from the technical resolution of the microarray platform by applying the former through software. This therefore presents a novel strategy for prenatal CMA that is a practical balance between leveraging the increased resolution of microarrays on the one hand, and the burden caused by variants of unknown significance on the other.

**Table 1:** Regions targeted for prenatal CMA.

Syndrome (OMIM ID)	Chromosome band
1p36 deletion (607872)	1p36
2q37 deletion (600430)	2q37
3q29 deletion (609425)/duplication (611936)	3q29
Wolf–Hirschhorn (194190)	4p16.3
Cri du Chat (123450)	5p15.2
Sotos (117550)	5q35.2q35.3
Williams–Beuren (194050)	7q11.23
8p23.1 deletion	8p23.1
Kleefstra (610253)	9q34.3
WAGR 11p13 deletion (194072)	11p13
Potocki–Shaffer (601224)	11p11.2
Angelman (105830)/Prader-Willi (176270)	15q11.2
15q24 deletion (613406)/duplication (613406)	15q24
16p13.3 deletion (610543)/duplication (613458)	16p13.3
Miller–Dieker (247200)/17p13.3 duplication (613215)	17p13.3
Hereditary Liability to Pressure Palsies (162500)/Charcot-Marie-Tooth type 1A (118220)	17p12
Smith–Magenis (182290)/Potocki–Lupski (610883)	17p11.2
17q11.2 deletion (613675)	17q11.2
Koolen–De Vries (610443)	17q21.31
Cat-Eye (115470)	22q11
DiGeorge (188400)/Velocardiofacial (192430)/22q11.2 duplication (608363)	22q11.2
Phelan–Mcdermid (606232)	22q13.33
Pelizaeus–Merzbacher (312080)	Xq22.2
Rett (312750)/Lubs X-Linked Mental Retardation (300260)	Xq28

We present the results from 342 prenatal samples, representing 20 months of diagnostic testing. We have anonymized and re-examined 249 of these samples, using our standard postnatal, full resolution analysis. The results of this retrospective re-analysis have allowed us to gauge the validity of the software-targeted approach, and to assess its advantages in terms of clinical utility, throughput and turnaround times.

## Materials and Methods

### Prenatal CMA testing strategy

**Figure 1:**
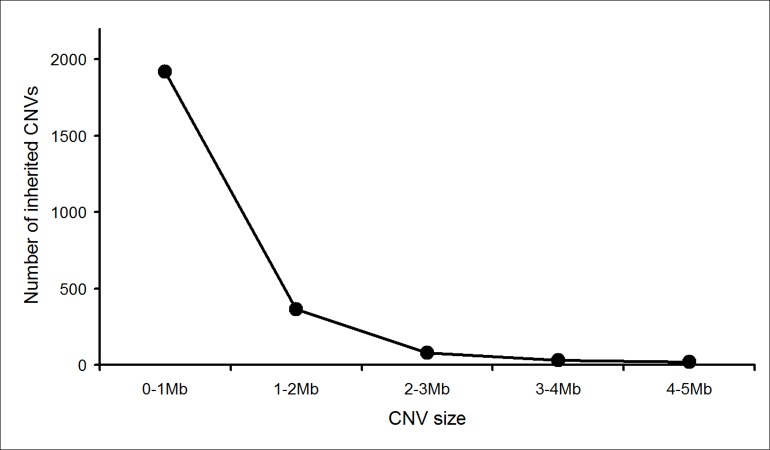
Size distribution of inherited CNVs detected by postnatal CMA.

The choice of 3 Mb as a cut-off for “calling” CNV outside the targeted regions was informed by our experience with results of postnatal CMA (>19,000 reported tests). Our data from postnatal samples showed that clinically benign CNVs were generally smaller in size and that they were also more likely to be inherited. Figure 1 shows the number of inherited CNVs when grouped by size. It indicates that using a 3 Mb backbone resolution for prenatal samples would exclude ∼97% of the inherited CNVs that would be detected if we were to use the full potential of our CMA platform (Table S1). This was desirable as we sought to minimize the uncertainty of prenatal CMA results.

We then examined the CNVs that would be reported at various backbone resolutions, excluding syndromic regions. Our postnatal data suggested that ∼1 in 5 CNVs reported with a 3 Mb backbone resolution would be inherited (Fig. S1). However, if we were to use a 2 Mb resolution CMA platform, this would increase to ∼1 in 3 non-syndromic CNVs being inherited. While our postnatal CMA experience informed us that the inheritance pattern of a CNV is not a perfect predictor of clinical significance and clear prognosis, we felt that the inheritance pattern was a useful proxy for these attributes here.

In addition to the low resolution backbone, we selected 29 regions associated with genetic syndromes that would have a clear prognosis, should they be detected in a fetus (see Table 1). These regions were not restricted by size as we designed a custom software module to detect CNVs >3 Mb and these deletion syndrome regions. Susceptibility loci such as 15q11.2 BP1–BP2, 1q21.1 (OMIM 612474 & 612475) and proximal 16p11.2 (OMIM 611913 & 614671) were excluded as they would be incidental to the ultrasound findings, would present interpretational difficulties and would be of little prognostic value.

As both the backbone resolution and the smaller, specifically targeted regions were configurable options in the software, this system was flexible and future-proof as we are free to change the resolution and add or remove targeted regions as our ability to interpret findings improved, or should we wish to refocus the test. While any such changes would require further validation, that process would be far simpler and more cost-effective than if we were using a fixed CMA platform.

### Diagnostic analysis

All prenatal samples received at our center receive QF-PCR testing to exclude trisomy for chromosomes 13, 18 and 21 (Hills et al., 2010). This test also detects triploidy, sex chromosome aneuploidies and the presence of maternal cell contamination at a level of 10% or greater. Those samples referred for fetal ultrasound anomalies (excluding soft markers for Down syndrome), where trisomies have been excluded, proceed to CMA by array CGH, using previously published protocols. Briefly, DNA was extracted from chorionic villi and amniotic fluid samples using the Puregene Tissue kit (Qiagen, UK), DNA was processed with the CGH Labeling Kit (Enzo Life Sciences, UK), unincorporated nucleotides were removed with the QIAquick Minelute kit (Qiagen, UK), and then hybridized to arrays following the manufacturer’s recommendations (Agilent, UK). Samples were incorporated into our postnatal array CGH pipeline, which paired two diagnostic samples, differentially labeled, together on a single Agilent oligonucleotide 60K array (AMADID 028469) (Ahn et al., 2013), thereby decreasing costs and increasing throughput. Prenatal samples were sexed by QF-PCR and then paired with clinically normal, sex-matched parental samples for postnatal patients that were undergoing inheritance studies.

Following quantification of fluorescence signals (Feature Extraction software, Agilent, UK), a combination of the ADM2 algorithm (threshold 6; Genomic Workbench, Agilent, UK) and a custom software module was used to detect CNVs >3 Mb and of specific deletion/duplication syndrome regions (see Table 1). These regions were chosen after consideration and discussion with clinical colleagues. If any CNVs were called by the software, the signal intensity across the CNV was assessed for the cyanine-3 and cyanine-5 labeled samples (relative to 5 other patient arrays on the same run showing no CNV in the called region) in order to confirm which sample carried the CNV as prenatal samples were hybridized with a second patient sample. This novel analysis method allowed confident identification of the abnormal sample on a paired array, as well as further cost and time savings. All genomic coordinates were based on the GRCh37 (hg19) reference genome annotation.

All CNVs were confirmed by either karyotyping or in situ hybridization studies, prior to reporting, in order to confirm sample identity (this was therefore a simple, targeted test rather than a comprehensive chromosome analysis); parental samples were requested where appropriate. No karyotype analysis was carried out for samples with no detected CNV.

Average reporting time for this cohort was 7 days, with a cost estimated to be approximately three-fifths that of culture and karyotyping of prenatal samples.

Occasionally, postnatal CMA was requested for newborns that had been tested prenatally. In these cases, the data from the prenatal CMA was re-analysed through the postnatal pipeline and a result issued without the need for a further test to be performed in the laboratory.

### Validation analysis

Array traces from 249 samples were anonymised, then re-analysed using a standard postnatal, 3-probe cut-off for CNV calling (Ahn et al., 2010). The size and gene content of any region called by the software was scrutinized to assess the likely clinical significance of the CNV.

## Results and Discussion

### Diagnostic prenatal CMA testing results

Following the strategy detailed in this paper, we have tested 342 prenatal samples. Of these, 23 (6.7%) samples were found to be abnormal (see Table 2) and were reported following confirmation of sample identity. All other samples were reported as “No Abnormality Detected”, with information on the size cut-off used and the deletion/duplication syndrome CNVs which had been excluded.

**Table 2:** Reported prenatal CMA results.

Reported result	Referral indication
1p36.32p36.12(4,481,264-22,855,001)x1	Nuchal 2.9 mm, cleft lip and palate, adjusted risk for trisomy 21 of 1/361, adjusted risk for trisomies 13 & 18 of 1/53.
1q21.1(145,413,387-145,747,269)x1^a^	Suspected TAR syndrome, absent or shortened radius & ulna bilaterally, bilateral radial aplasia with shortened phalanges and di-phalyngeal thumbs, both hands acutely abducted, both ulnae are approx 2/3 the normal length, humeri on the 5th centile, long bones of the lower limbs are on the 50th centile.
3p21.31(45,266,030-48,311,229)x3	Known familial insertion of chr3 material into another chromosome.
3p24.1p22.3(30,485,387-34,962,363)x1	Bilateral borderline ventriculomegaly, absent cavum septum pellucidum.
4p16.3p14(72,446-35,935,983)x1	Nuchal 3.1 mm.
4p16.3p16.1(514,449-8,667,610)x1	Bilateral talipes, cleft lip, single umbilical artery.
4p15.33p15.32(13,625,716-17,418,852)x1	Interuterine growth retardation, low PAPPA and combined tests.
4p15.31p15.1(20,541,127-28,451,250)x1	Nuchal 2.0 mm, exomphalos, absent nasal bone, reverse ductus.
6p25.3p22.2(259,527-25,416,824)amp	Mild ventriculomegaly, potential brain abnormality.
6q23.2q24.2(135,056,331-143,515,719)x1	Oligohydramnios, echogenic bowel.
9p24.3(214,366-2,197,859)x1, 9p24.2p13.1(2,418,074-40,508,819)x3	Bilateral genu recurvatum, moderate ventriculomegaly.
9p24.3p13.1(214,366-40,508,819)x4	Nuchal 8.3 mm.
9q33.3q34.3(126,795,265-141,008,915)x3	Muscular ventral-septal defect, tricuspid regurgitation, abnormally shaped aortic arch.
10q11.22q11.23(46,951,236-52,004,151)x1	Bilateral ventriculomegaly.
11q23.3q25(116,693,628-134,446,160)x3, 22q11.1q11.21(16,053,472-20,311,763)x3	Possible micrognathia with prominent upper lip, brain cyst, dilated 3rd ventricle, stomach not clearly insulated, complete or partial agenisis of corpus callosum, deficient cerebellar vermis.
13q31.3q34(94,493,888-115,059,020)x1	High risk for trisomy 13, holoprosencephaly, diaphragmatic hernia, cleft palate
15q21.2q25.1(51,740,270-79,762,418)x3	Shortened femur, bilateral hydronephrosis, interuterine growth retardation, 2x vessel cord, dysmorphic, downslanting palpebral fissures, low set ears.
17p11.2(16,532,735-20,221,695)x3	Long bones around or below 3rd centile (including femur), dilation of the intra-abdominal portion of the umbilical vein (varix).
22q11.1q13.33(17,096,854-51,178,264)x3	Nuchal 4.4 mm, trisomy 21 risk of 1:61.
22q11.21(18,896,971-21,440,514)x1	Nuchal 4.2 mm, trisomy 21 risk of 1:70.
22q11.21(18,896,971-21,801,661)x3	Ventriculomegaly, hydronephrosis, shortening of the long bones.
22q13.31q13.33(45,576,756-51,178,264)x1	Echogenic bowel, urinary tract/renal anomaly.
Xp11.3p11.21(44,307,282-58,051,765)x1∼2	Intrauterine growth retardation, talipes, stomach not visible on ultrasound.

**Notes.**

a This fetus was referred for suspected TAR syndrome and therefore an additional targeted region was added for RBM8A.

All genomic coordinates are for human genome reference version hg19/GRCh37.

Our software targeted system provides flexibility to make changes “live”, in response to information on specific cases. For example, in one of our cases (indicated by an asterix in Table 2), the fetus was reported to have ultrasound anomalies suggestive of thrombocytopenia-absent radius syndrome (OMIM 27400). An additional targeted region was therefore added to the analysis pipeline and the copy number of RBM8A could therefore be analyzed. The array showed deletion of this gene, consistent with the referral indication and this finding was reported. This type of adaptive testing has the potential to be hugely effective in a prenatal setting.

### Retrospective reanalysis results

Retrospectively, 249 of the prenatal samples were anonymised and reanalysed at full resolution, using a 3-probe cut-off as for our postnatal samples. A further 46 CNVs were detected in 44 of these samples (17.7%). These CNVs ranged in size from 3 kb to 2 Mb for deletions (see Table 3) and 0.5 kb to 2.5 Mb for increased copy number (see Table 4). There were only two CNVs between 2 and 3 Mb in size. The size and gene content of all 46 CNVs indicated that none of them was likely to be of clear clinical significance. A few CNVs included genes/loci that had some association with a clinical phenotype (e.g., PAX3, SLC9A9, and susceptibility loci such as 15q11.2 BP1-BP2), but none of these had a clear prognosis and thus would have presented the clinicians with difficult counseling issues and couples with difficult decisions, a situation which our prenatal CMA testing strategy was designed to avoid.

**Table 3:** Further deletion CNVs uncovered by retrospective reanalysis of prenatal CMA data at full resolution.

Deletion CNV	Referral indication
1q21.1(145,413,387-145,747,269)x1	Nuchal 4.5 mm.
2p21(44,507,914-44,531,188)x1	Brain ventricle/hemisphere >97th centile, trisomy 21 risk of 1:13.
2p16.3(50,881,995-50,947,729)x1	Complete transposition of the great arteries.
2p12(75,347,691-75,729,632)x1	Tricuspid valve dysplasia, fetal hydrops.
2q35(220,096,681-220,116,241)x1	Nuchal 1.6 mm, absent ductus venous, trisomy 21 risk of 1:19121, trisomy 13/18 risk of 1:35067.
2q36.1(222,834,667-224,926,273)x1^a^	Nuchal 4.1 mm, trisomy 21 risk of 1:112.
3p24.3(20,021,595-20,052,991)x1	Nuchal 1.5 mm, echogenic bowel, liver anomaly.
4q24(107,063,807-107,248,637)x1	Isolated aberrant right subclavian artery.
6p22.2(26,440,746-26,463,502)x1	Tetralogy of Fallot, small for gestational age (<10th centile).
8q23.3(113,630,231-113,960,067)x1	Nuchal 4.1 mm.
10q26.3(135,352,371-135,372,492)x1	Nuchal 2.5 mm, trisomy 21 risk of 1:2005, stomach on right side, suspected arterioventricular defect.
11q22.1(97,762,150-98,228,688)x1	Trisomy 21 risk of 1:8.
14q24.3(76,352,571-76,522,811)x1	Nuchal 5 mm.
15q11.2(22,318,596-23,085,096)x1	Severe growth restriction, oligohydramnios.
15q11.2(22,765,627-23,085,096)x1	Aberrant right subclavian artery.
15q11.2(22,765,627-23,085,096)x1	Left-sided diaphragmatic hernia.
16q23.2(81,293,283-81,367,334)x1	Nuchal 5.7 mm, Trisomy 21 risk of 1:204, Trisomy 13/18 risk of 1:129.
19p13.2(7,070,409-7,168,093)x1^b^	Short long bones, know early pregnancy haematoma (sub chorionic bleeding).
19q13.2(42,263,338-42,289,030)x1	Nuchal 3.2 mm.
22q11.23(23,627,338-24,040,236)x1^b^	Short long bones, know early pregnancy haematoma (sub chorionic bleeding).
Xp22.33(1,378,590-1,689,610)x1	Borderline ventriculomegaly.
Xp22.33(2,066,580-2,343,577)x1	Nuchal 4.4 mm.
Xp22.11(23,018,416-23,021,667)x1	Nuchal 3.1 mm, abdominal cyst, crown–rump length small for gestational age, trisomy 21 risk of 1:37.

**Notes.**

a PAX3 is deleted, which is indicative of Waardenburg syndrome, Type I.

b These two CNVs were carried by a single fetus.

All genomic coordinates are for human genome reference version hg19/GRCh37.

**Table 4:** Further amplification CNVs uncovered by retrospective reanalysis of prenatal CMA data at full resolution.

Amplification CNV	Referral indication
2p16.3(50,625,488-51,057,883)x3	Echogenic bowel.
2q11.2(98,019,585-98,274,335)x3	Nuchal 1.0 mm, omphalocele.
3q22.2(134,204,455-134,204,970)x4	Atrioventricular septal defect, coarctation of aorta.
3q24(142,840,204-143,579,847)x3	Nuchal >4 mm.
4q24(102,735,053-102,897,983)x3	Polyhydramnios, ventriculomegaly, bilateral talipes, suspected Charcot-Marie-Tooth syndrome.
6p12.3(50,153,611-50,519,464)x3	Nuchal 5.2 mm.
6q21(105,548,868-107,397,152)x3	Polyhydramnios, pleural effusion, hydronephrosis.
6q21(111,067,339-111,478,900)x3	Hypoplastic left heart syndrome, complex congenital heart disease, interstinal malrotation, suspected 22q11.2 deletion syndrome.
8p12(33,210,383-33,455,764)x3	Fetal cardiac abnormality.
10q21.3(64,902,960-67,399,362)x3	Coarctation of aorta, large ventricular septal defect, suspected 22q11.2 deletion syndrome.
12p12.1(21,615,645-21,689,158)x3	Nuchal 2.3 mm, double outlet right ventricle, spontaneous rupture of membranes.
14q11.2(22,323,878-22,964,922)x3	Nuchal 4.3 mm, trisomy 21 risk of 1:10.
14q11.2(22,669,442-22,964,922)x3	Intrauterine growth retardation.
19p13.2-p13.13(13,865,337-13,933,080)x3	Nuchal 3.8 mm.
Xp22.33(658,210-1,259,140)x3	Bilateral talipes.
Xp22.33(919,416-1,259,140)x3^a^	Nuchal 1.9 mm, cardiac abnormality.
Xp22.33(970,702-2,017,358)x3	Polyhydramnios, trisomy 21 risk of 1:400.
Xp22.33(1,217,016-1,378,646)x3	Aberrant right subclavian artery, suspected 22q11.2 deletion syndrome.
Xp22.33(1,314,735-1,347,344)x3	Cardiac anomaly, ventriculomegaly.
Xp22.33(1,755,741-2,017,358)x3	Hydrops.
Xp22.31(6,551,154-8,032,120)x3	Bilateral talipes.
Xp22.2(16,147,216-16,809,305)x2	Nuchal 4.7 mm, trisomy 13 risk of 1:82, trisomy 18 risk of 1:59.
Xq28(154,133,237-154,560,375)x2^a^	Nuchal 1.9 mm, cardiac abnormality.

**Notes.**

a These two CNVs were carried by a single fetus.

All genomic coordinates are for human genome reference version hg19/GRCh37.

**Figure 2:**
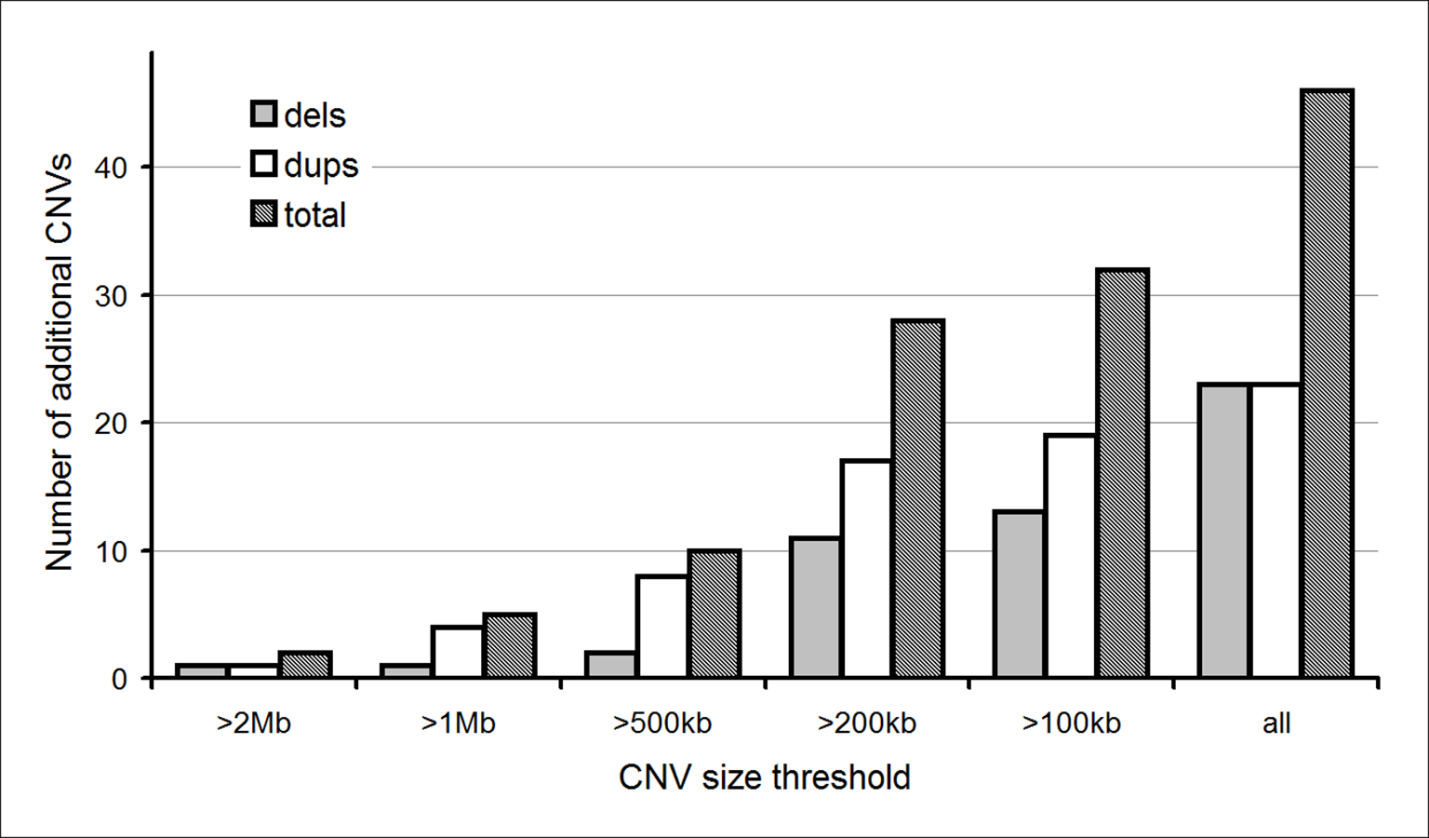
Additional CNVs detected if backbone resolution of prenatal CMA is increased.

Figure 2 shows the effect of increasing backbone resolution in terms of the increased number of CNVs that would have been detected for our reanalyzed prenatal cohort. Our reanalysis has shown that lowering the threshold would greatly increase the time and associated costs, with no added clinical utility in this cohort. Furthermore, these additional findings would have potentially caused anxiety whilst inheritance studies were in progress, and further into the pregnancy and early years of the resulting progeny; the possibility of an unnecessary pregnancy termination should also be considered.

## Conclusion

Due to the complexity of the issues surrounding prenatal CMA, and different financial models for healthcare delivery, it may only be possible to reach consensus methodology at a national level, and even that has been elusive to date. It is generally agreed that CMA should be used for prenatal detection of CNV, as this test has a higher resolution than traditional G-banded karyotype analysis and can thus identify clinically important CNV not previously detectable. However, pregnant women are vulnerable and anxious, and therefore an ideal prenatal test should provide results in the shortest possible timeframe, and these results should be clear-cut and should provide straightforward choices for the couples. Findings of uncertain significance and “toxic knowledge” associated with incidental findings of clinical significance but of no relevance to the fetal anomalies, cause distress and anxiety not only throughout the pregnancy, but beyond (Bernhardt et al., 2013); ambiguous results and incidental findings are therefore considered by some to present ethical dilemmas that conflict with clinical principles of non-maleficence (Mikhaelian et al., 2013; Stark et al., 2013). CMA platforms have therefore been designed and implemented that target regions associated with deletion/duplication syndromes and that have a low resolution “backbone” with widely spaced probes (Fiorentino et al., 2011; Park et al., 2010); these platforms detect syndrome-associated deletions and duplications, and provide information on large CNVs across the rest of the genome. However, they are inflexible, as new platforms must be developed each time a new region of clinical significance emerges.

More recently, centers have used higher density arrays, assigning CNVs to various categories, with different reporting frameworks for each category (Brady et al., 2013; Ganesamoorthy et al., 2013; Vetro et al., 2012). The importance of discussion between laboratory and clinician before assigning a CNV to a specific category is emphasized (Liao et al., 2013; Vetro et al., 2012), with one study suggesting a pan-center committee for scrutinizing CNVs and making recommendations (http://www.nets.nihr.ac.uk/projects/eme/106003). However, these approaches involve delays in reporting until scrutiny and discussion is complete, and the complexity of the decision-making process increases the cost of the test. In addition, the possession of information that will not be conveyed to the couple could be considered an infringement of their right to autonomy.

The software-targeting approach described in this paper has three key advantages over previously described prenatal CMA strategies. Firstly, it allows far greater test adaptability than hardware-restricted platforms, and thus permits the incorporation of new clinically significant regions (e.g., emergent syndromes), or changes in backbone resolution. Second, it provides the ability to personalize the prenatal CMA test to an individual clinical presentation, for instance as demonstrated in the example above of a fetus suspected to have TAR syndrome; it is for situations such as this that some laboratories have adopted high resolution CMA. Finally, and most importantly, the problems associated with incidental findings and CNVs of uncertain significance are minimized, as is the anxiety for the pregnant woman and her partner.

The results of the anonymization of array traces from our cohort showed that no CNVs of serious clinical significance were unreported (see Tables 3 and 4). There were only two CNVs between 2 Mb and 3 Mb. One was an ∼2.5 Mb duplication of material from the long arm of chromosome 10 (64,902,961–67,399,362bp), that contained no genes associated with any clinical phenotype. The other CNV was an ∼2 Mb deletion in the long arm of chromosome 2 (indicated by an asterix in Table 3); this region includes the PAX3 gene, deletion of which is causative of Waardenburg syndrome, Type 1 (OMIM 193500). The main features of this syndrome are dystopia canthorum, deafness, white forelock, and ocular and skin pigmentation anomalies, although the penetrance of these features is variable (DeStefano et al., 1998). Interestingly, while this manuscript has been in preparation, a neonate has been referred to our center with facial dysmorphism and poor feeding. The prenatal test, carried out using our strategy following a nuchal translucency measurement of 4.1 mm, had reported “no abnormality detected”; the referring clinician was now requesting a full CMA result. Unmasking of the prenatal trace showed that this was the case with the PAX3 deletion described above. The child has passed a newborn hearing screen and apart from mild facial dysmorphism shows no other clinical phenotype to date.

We feel that these two cases provide support for using a 3 Mb threshold, rather than reducing the size threshold to 2 Mb, as one was not clinically significant and prenatal reporting of the other CNV would have caused serious counseling difficulties, due not only to the nature of the clinical features, but also to uncertainty as to the severity of the phenotype; any such reporting would be likely to cause distress and uncertainty for the pregnant woman and her partner, and would have presented them with extremely difficult decisions on the future of the pregnancy. There are certainly CNVs between 2 Mb and 3 Mb that are clinically significant with a clear prognosis, however, our postnatal data informs us that after excluding the syndromes that are included on our prenatal platform, only 8% (CI 95% [5–13%]) of CNVs that are 2–3 Mb in size were interpreted as clearly pathogenic. This suggests that the vast majority of non-syndromic CNVs in this size range are likely to be of unclear clinical significance.

Although we have described a set of regions to target and a backbone resolution, other laboratories choosing the CMA approach described here should determine their own threshold and targeted regions. Perhaps more appropriately, best practice guidelines could be produced based on national consensus; these could be reviewed periodically and updated to incorporate advances in the field.

One limitation of CMA is the inability to detect balanced chromosome rearrangements. These are reported to occur in <1% of tested pregnancies (Giardino et al., 2009) and if they are inherited, then there is considered to be no risk to the fetus’ health. Currently, there is little data available for the pathogenicity of *de novo* balanced rearrangements as most of the literature is based on karyotype results, and many apparently balanced translocations and inversions are not truly balanced when examined with a higher resolution technique. Whilst offering karyotyping in addition to CMA would overcome this limitation, “double testing” of these samples is not financially viable under UK healthcare provision. CMA is therefore offered at our centre as the first-line genome-wide prenatal test for pregnancies with structural abnormalities detected by ultrasound and/or raised nuchal translucency measurements (>3 mm), this is clearly the best option for our patients.

For severe fetal phenotypes detected on ultrasound scan, a decision to terminate may be based on the ultrasound findings alone; in these cases, the CMA result will have value in determining the etiology and recurrence risk for any causative CNVs detected. For milder phenotypes, such as a heart defect or isolated raised nuchal thickness, a normal CMA result will provide reassurance that the ultrasound finding is not due to a deletion syndrome with associated neurodisability. CNV of syndrome regions currently targeted by our software, shown in Table 1, is of known pathogenic effect, although in some cases there may be variation in severity. For instance, although the phenotype associated with the “common” 22q11.2 deletion is generally severe, the features associated with the reciprocal duplication are very variable and relatively mild. Unfortunately, even with the targeted approach described here, it was not possible to avoid detecting these duplications, as this region is targeted. However, we are now developing the software further to differentiate between reduced and increased copy number, and so will be able to increase further the selectivity of this test.

Many deletion syndromes would not be expected to produce abnormal features detectable by ultrasound; for this reason, the possibility of using array CGH to test all prenatal samples has been raised (Brady et al., 2013). The objections to this are generally based on the concomitant detection of CNVs and incidental findings, with the associated problems discussed above. A software-targeted approach as described here would circumvent these concerns, and could provide exclusion of deletion syndromes for all pregnancies undergoing prenatal sampling.

Sequencing approaches for the prenatal detection of fetal CNV using free fetal DNA in maternal blood samples have recently become available for detection of the common trisomies (Chiu et al., 2011; Jiang et al., 2012; Palomaki et al., 2011); these approaches are currently expensive, and may take around two weeks to report. Until this technology becomes cheaper, results available more rapidly, and CNVs accurately detected across the genome, CMA will continue to be an important tool in obstetric practice, and should become the standard of care at all centers. The approach described in this paper, backed by international and national guidelines on size cut-offs and regions to be targeted, should allow the rapid introduction of this test for the benefit of all women having prenatal diagnosis.

## Supplemental Information

10.7717/peerj.354/supp-1 Fig. S1 Inheritance pattern of CNVs detected by postnatal CMA if various backbone resolutions are applied

10.7717/peerj.354/supp-2 Table S1 Size distribution of *de novo* and inherited CNVs detected by postnatal CMA

10.7717/peerj.354/supp-3 Supplemental Information 3 Raw data
