# Supplementary figures and images for: A new direction for prenatal chromosome microarray testing: software-targeting for detection of clinically significant chromosome imbalance without equivocal findings
Source: PeerJ. 2014 Apr 22;2:e354. doi: 10.7717/peerj.354 (PMC4006225; doi:10.7717/peerj.354)

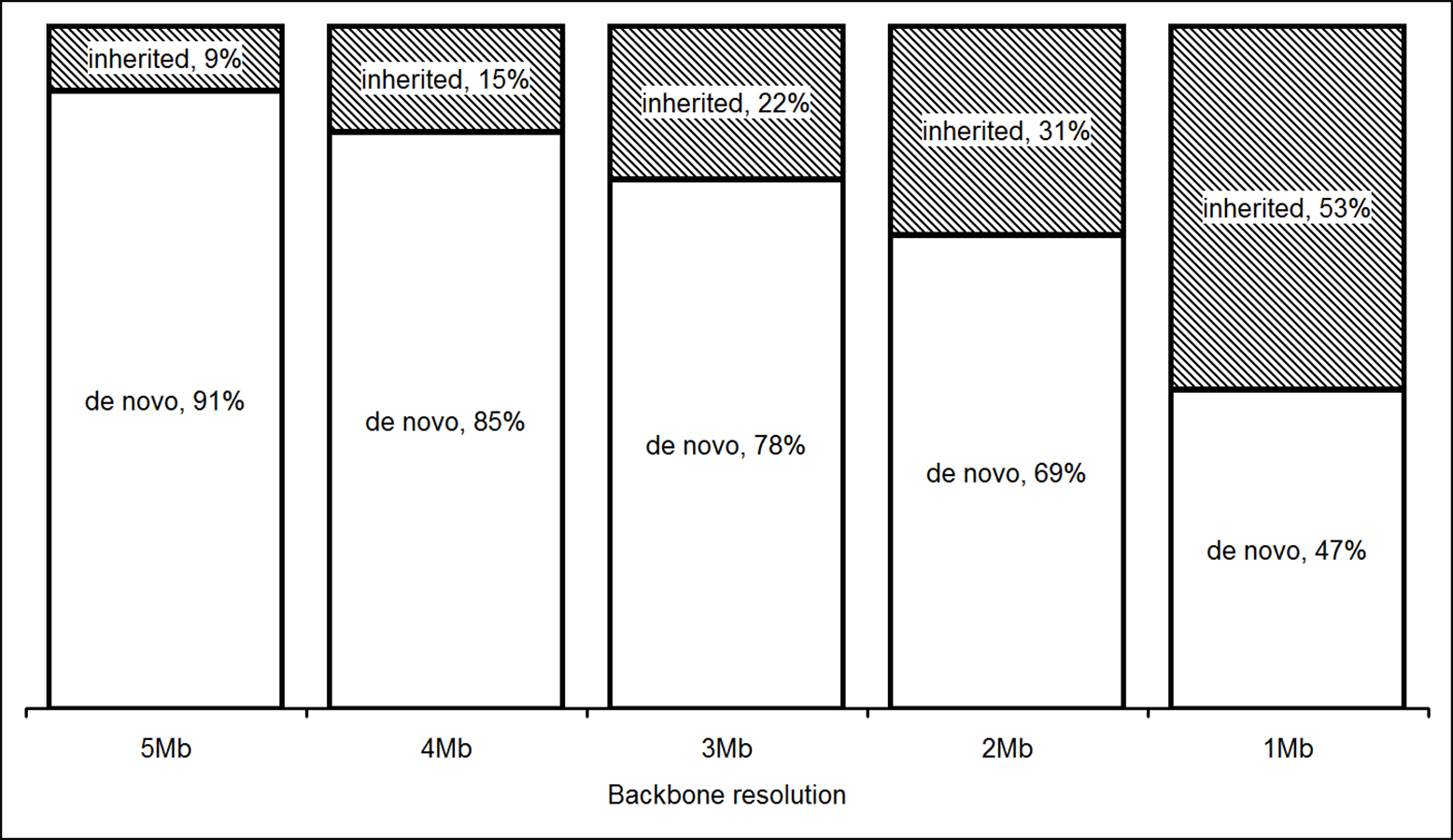

Supplement: Fig. S1 [file peerj-02-354-s001.png]
