# Supplementary material for: A new direction for prenatal chromosome microarray testing: software-targeting for detection of clinically significant chromosome imbalance without equivocal findings
Source: PeerJ. 2014 Apr 22;2:e354. doi: 10.7717/peerj.354 (PMC4006225; doi:10.7717/peerj.354)
Supplement: Table S1 [file peerj-02-354-s002.doc]

**SUPPLEMENTARY INFORMATION**

Table S1. Size distribution of *de novo* and inherited CNVs detected by postnatal CMA.

| CNV size | Number of *de novo* CNVs | Number of inherited CNVs |
| --- | --- | --- |
| 0 - 1Mb | 230 | 1918 |
| 1 - 2Mb | 115 | 362 |
| 2 - 3Mb | 101 | 79 |
| 3 - 4Mb | 37 | 31 |
| 4 - 5Mb | 28 | 18 |
| 5Mb + | 163 | 17 |
